# Increased Aqueous Humor GDF15 Is Associated with Worse Visual Field Loss in Pseudoexfoliative Glaucoma Patients

**DOI:** 10.1167/tvst.9.10.16

**Published:** 2020-09-15

**Authors:** Jonathan B. Lin, Arsham Sheybani, Andrea Santeford, Alicia De Maria, Rajendra S. Apte

**Affiliations:** 1 Department of Ophthalmology and Visual Sciences, Washington University in St. Louis, St. Louis, MO, USA; 2 Department of Developmental Biology, Washington University in St. Louis, St. Louis, MO, USA; 3 Department of Medicine, Washington University in St. Louis, St. Louis, MO, USA

**Keywords:** GDF15, pseudoexfoliative glaucoma, neurodegeneration, biomarkers

## Abstract

**Purpose:**

To determine whether increased growth differentiation factor 15 (GDF15) in aqueous humor (AH) is associated with worse visual field loss in patients with pseudoexfoliative glaucoma (PXG).

**Methods:**

We recruited 12 patients (6 males, 6 females) with primary open-angle glaucoma (POAG) or PXG who were scheduled to undergo glaucoma surgery. AH was obtained from the initial peripheral paracentesis for the planned glaucoma surgery, and GDF15 levels were quantified with enzyme-linked immunosorbent assay by an investigator masked to clinical information. Humphrey visual field testing was performed as a part of routine care; results were obtained by reviewing the medical record.

**Results:**

AH GDF15 was detectable in patients with POAG and PXG. Increased AH GDF15 was significantly associated with worse mean deviation in patients with POAG (*r* = −0.94; 95% confidence interval [CI], −0.99 to −0.33; *P* = 0.02) and PXG (*r* = −0.92; 95% CI, −0.99 to −0.41; *P* = 0.01).

**Conclusions:**

AH GDF15 is detectable in patients with PXG and POAG. Elevated AH GDF15 is strongly associated with worse mean deviation in both subgroups. These findings suggest that GDF15 may be a molecular marker of glaucoma severity that is generalizable to multiple types of glaucoma regardless of the underlying etiology.

**Translational Relevance:**

This study provides proof of concept that GDF15, a molecular marker of retinal ganglion stress that was initially identified in rodent models, may have clinical utility as a measure of glaucoma severity not only in POAG but also in PXG.

## Introduction

Glaucoma is a family of diseases characterized by neurodegeneration of retinal ganglion cells (RGCs). The most common cause of secondary, open-angle glaucoma is pseudoexfoliative glaucoma (PXG), caused by deposition of amyloid-like fibrillary material in the trabecular meshwork and consequent obstruction of aqueous humor (AH) outflow. Compared to primary open-angle glaucoma (POAG), PXG exhibits a more serious clinical course, characterized by higher intraocular pressure (IOP) at the time of diagnosis, faster visual field progression, poorer response to medical therapy, and increased need for surgical intervention.^1^^–^^5^

Similar to managing POAG, one major challenge of managing PXG is the inability to monitor RGC neurodegeneration directly. Instead, clinicians rely on surrogate measurements of RGC health and function, such as cup-to-disk ratio, nerve fiber layer thickness, and visual field testing, and known risk factors of glaucomatous progression, such as IOP, to guide clinical decisions.^6^ Therefore, there is a clinical need for novel molecular markers that directly measure RGC stress that could be used to guide treatment decisions in glaucoma, especially in PXG.

We recently discovered that growth differentiation factor 15 (GDF15), a member of the transforming growth factor β superfamily, is a molecular marker of retinal ganglion stress in rodent models.^7^ Furthermore, we found that GDF15 was elevated in the AH of patients with POAG with levels increasing stepwise with worse visual field loss by Hodapp-Parrish-Anderson staging.^7^ However, since we tested only patients with POAG, we were unable to determine whether these findings are generalizable to other types of glaucoma.

In this single-center, cross-sectional study, we sought to determine whether AH GDF15 is also detectable in PXG and whether it also increases with worse visual field loss in PXG. Since GDF15 is a molecular marker of retinal ganglion stress in rodents, we hypothesized that we would observe similar levels of AH GDF15 in patients with PXG as we have previously reported for patients with POAG. These findings would support the possibility of using GDF15 as a molecular marker of glaucoma severity in multiple forms of glaucoma beyond POAG.

## Methods

To determine the appropriate sample size to identify a significant association between AH GDF15 and mean deviation, we performed a power analysis using G*Power 3.1.9.2.^8^ Estimating a correlation coefficient between AH GDF15 and mean deviation of *r* = 0.75 based on our previous data, we calculated a sample size of *N* = 9 to achieve 80% power at a two-tailed alpha of 0.05. To account for the possible need to exclude some patients, we recruited 12 participants from Washington University in St. Louis who were treated by two glaucoma surgeons. Patients were included if they had POAG or PXG and were determined to be candidates for combined cataract extraction with intraocular lens implantation and glaucoma surgery of any type as part of their usual care. Glaucoma subtype was determined by the treating physician based on physical exam findings, visual field testing, optic nerve head imaging, and gonioscopy, which were performed in the clinic as a part of routine care. Eyes were excluded if there was active inflammatory eye disease, any retinopathy, optic nerve degeneration from nonglaucomatous causes, or any condition that would preclude Humphrey visual field (HVF) testing. In the final analysis, we excluded one patient since it was determined during retrospective chart review that the patient had a history of ocular inflammation with hand-motion vision in the study eye, giving us a final study sample of *N* = 11.

This study was approved by the Institutional Review Board of the Human Research Protection Office of Washington University in St. Louis. All procedures adhered to the tenets of the Declaration of Helsinki. All patients provided written informed consent after explanation of the nature and possible consequences of the study. One AH sample was obtained for each patient in the operating room during the initial steps of the planned glaucoma surgery as previously described.^7^ Briefly, a blunt cannula on a tuberculin syringe was inserted into the initial peripheral paracentesis and used to remove 50 to 100 µL AH. The glaucoma surgery was then performed. Meanwhile, AH was immediately placed on dry ice and stored at −80°C until further analysis. GDF15 levels were measured using the human GDF15 Quantikine enzyme-linked immunosorbent assay (ELISA) kit (R&D Systems) with quantification by comparison to a standard curve with four-parameter logistic regression. The investigator (A. Santeford) measuring GDF15 levels was masked to clinical information. All GDF15 levels were measured on the same ELISA plate to minimize interplate variability.

Demographic and clinical information, including presurgical baseline IOP, presurgical number of medication classes, presurgical cup-to-disc ratio, presurgical retinal nerve fiber layer (RNFL) thickness, and presurgical mean deviation by HVF, were obtained by retrospective chart review by an investigator masked to GDF15 levels (JBL). If a participant had multiple HVF results available, the one that was obtained closest in time to the AH collection date was used. We performed statistical analysis and data visualization using R version 3.6.2 and RStudio version 1.2.5003. To compare means, we used the Mann-Whitney *U* test for two groups or Kruskal-Wallis one-way analysis of variance for three groups. We performed post hoc testing with pairwise Wilcoxon rank-sum tests, with Bonferroni-adjusted *P* values to account for multiple comparisons. To determine relationships between categorical variables, we used the Fisher exact test; to determine associations between continuous variables, we calculated Pearson correlation coefficients and generated linear regression models. Due to the small sample, we also calculated Spearman rank correlation coefficients to confirm the robustness of our results. We also performed sensitivity analysis by reanalyzing data without high-leverage values to confirm the robustness of our results. Where indicated, we also performed some analysis of the data in the present article alongside data we have previously published.^7^ We considered *P* < 0.05 to be statistically significant.

## Results

Demographic and clinical characteristics of the participants are shown in the Table. There were six male and six female participants. There were no differences in the sex, age, study eye laterality, presurgical baseline IOP, presurgical number of medication classes, presurgical cup-to-disc ratios, presurgical RNFL thickness, or presurgical mean deviation (MD) between patients with POAG or PXG. After AH was obtained, study participants underwent a variety of glaucoma surgeries, including ab interno goniotomy, gonioscopy-assisted transluminal trabeculotomy, Kahook Dual Blade goniotomy (New World Medical, Rancho Cucamonga, CA), Hydrus microstent placement (Ivantis, Irvine, CA), XEN gel stent placement (AqueSys, Taipei, Taiwan), or Ahmed glaucoma valve placement (New World Medical). There was no significant difference in the types of glaucoma surgeries that patients with POAG or PXG underwent. None of the patients with POAG had a prior glaucoma surgery; two of the patients with PXG had previous selective laser trabeculoplasty (SLT), and one patient with PXG had previous SLT and Ahmed glaucoma valve placement.

**Table.** Demographic and Clinical Characteristics of Study Participants

Characteristic	POAG	PXG	*P* Value
Sex, No.^a^ (%)			0.57^b^
Male	4 (66.7)	2 (33.3)	
Female	2 (33.3)	4 (66.7)	
Age, mean (SD)	67.8 (5.8)	73.8 (6.9)	0.13^c^
Study eye, No. (%)			1.00^b^
Oculus dexter	4 (66.7)	3 (50.0)	
Oculus sinister	2 (33.3)	3 (50.0)	
Planned glaucoma surgery,^d^ No. (%)			0.42^b^
Ab interno goniotomy	2 (33.3)	1 (16.7)	
Gonioscopy-assisted transluminal trabeculotomy	1 (16.7)	2 (33.3)	
Kahook Dual Blade goniotomy	0 (0.0)	3 (50.0)	
Hydrus microstent	1 (16.7)	0 (0.0)	
XEN gel stent	1 (16.7)	0 (0.0)	
Ahmed glaucoma valve	1 (16.7)	0 (0.0)	
Presurgical baseline IOP, mean (SD)	19.0 (8.0)	20.8 (7.3)	0.58^c^
Presurgical classes of medications,^e^ mean (SD)	2.8 (1.9)	2.7 (0.8)	0.78^c^
Presurgical cup-to-disc ratio, mean (SD)	0.74 (0.19)	0.77 (0.23)	0.85^c^
Presurgical RNFL thickness,^f^ mean (SD)	66.5 (20.6)	62.0 (20.2)	1.00^c^
Presurgical MD, mean (SD)	−8.9 (7.3)	−10.5 (13.5)	0.66^c^

a Number of participants in each category.

b Not statistically significant by Fisher exact test.

c Not statistically significant by Mann-Whitney *U* test.

d All participants also had cataract extraction with intraocular lens placement.

e Oral carbonic anhydrase inhibitors were considered a separate class.

f Data were not available from one patient with POAG and two patients with PXG.

**Figure 1.**
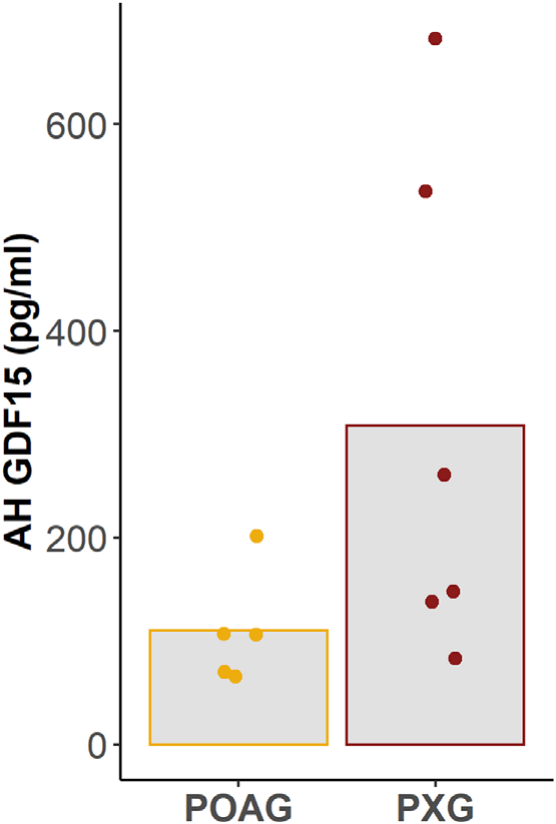
AH GDF15 was detectable in patients with POAG and those with PXG. *Height of bars* represents mean; *filled circles* represent individual patients.

**Figure 2.**
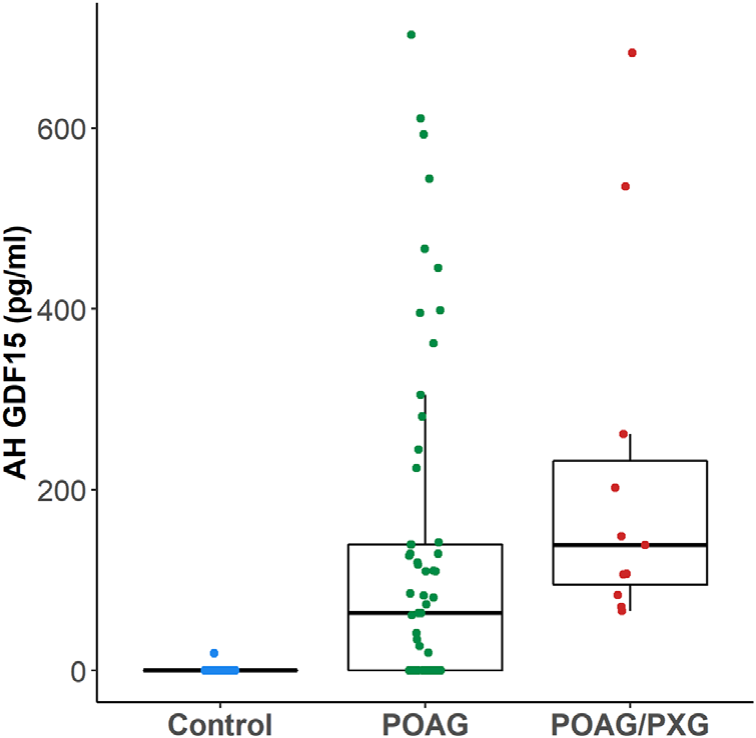
There was a significant difference in AH GDF15 levels when comparing the patients with POAG and PXG in this study (“POAG/PXG”; *right column* in *red*) to historical patients with POAG (“POAG”; *middle column* in *green*) and historical healthy patients without glaucoma (“Control”; *left column* in *blue*), which are both from Ban et al.^7^ (Kruskal-Wallis one-way analysis of variance; *P* < 0.001). Post hoc testing by pairwise Wilcoxon rank-sum tests with Bonferroni-adjusted *P* values to account for multiple comparisons showed that there was a significant difference between POAG/PXG and control (*P* < 0.001) and between POAG and control (*P* < 0.001). There was no significant difference between POAG/PXG and POAG (*P* = 0.084). *Filled circles* represent individual patients.

AH GDF15 was detectable in patients with POAG and PXG (Fig. 1). Although we did not recruit healthy patients without glaucoma in this study, these AH GDF15 levels were significantly elevated compared to a historical control group of patients without glaucoma, which we published previously (*P* < 0.001; Fig. 2). In fact, in this past study, ∼88% of patients without glaucoma had AH GDF15 levels that were below the limit of detection of commercially available ELISA.

**Figure 3.**
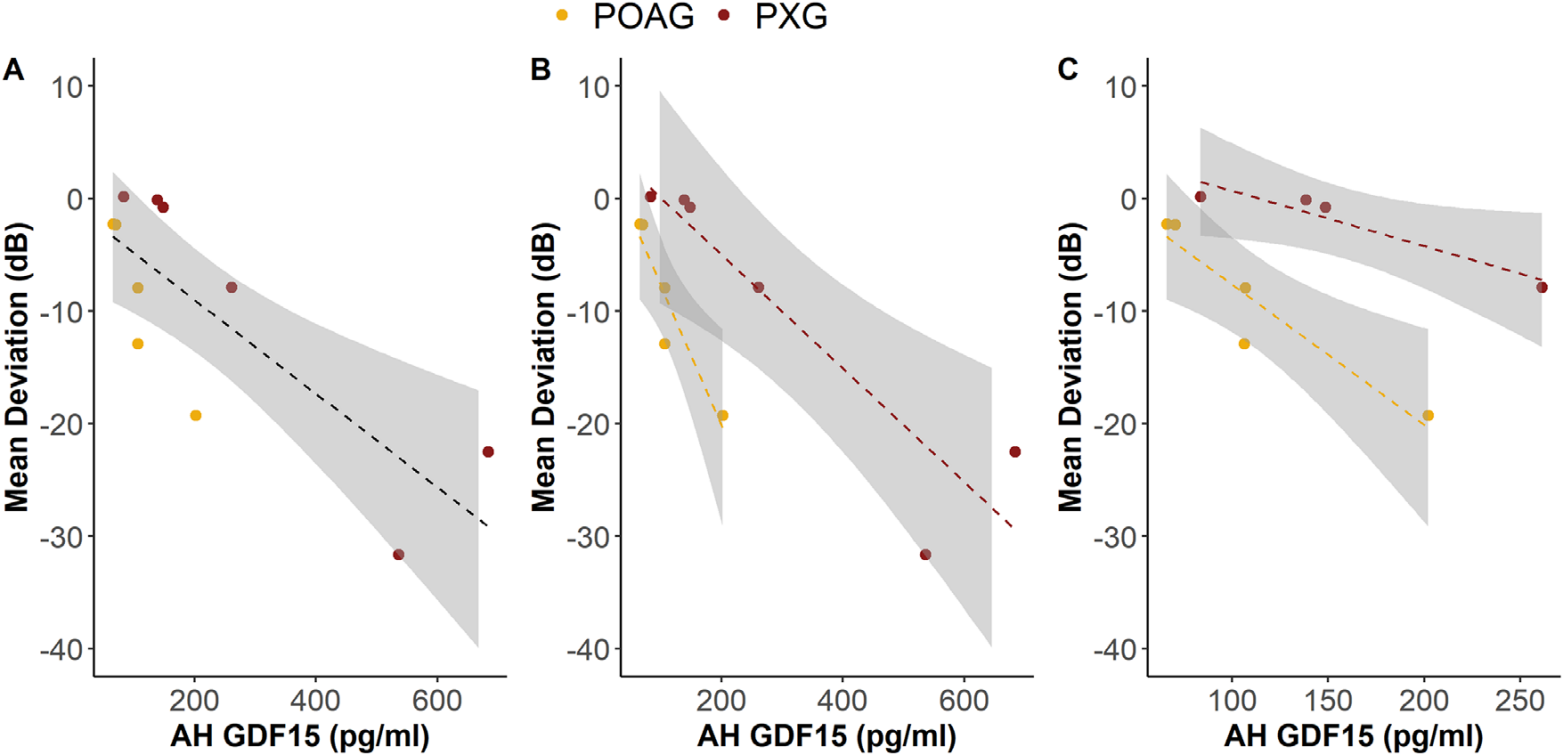
(A) There was a strong negative association between AH GDF15 and mean deviation on Humphrey visual field testing when patients with POAG and PXG were analyzed together. (B) There was a similar negative association when analyzing patients with POAG and PXG separately. (C) The negative association remained significant when reanalyzing the data without the two high-leverage patients with PXG, strengthening the robustness of our findings. *Dashed lines* indicate lines of best fit; *shaded regions* indicate 95% confidence interval bands; *filled circles* represent individual patients.

To determine whether AH GDF15 levels increase stepwise with worse visual field loss, we also compared the relationship between AH GDF15 and severity of visual field loss between patients with POAG and PXG. For all patients, HVF results obtained through routine clinical care were available within a median of 0.19 years from the day of AH collection (range, 0.033 to 2.41 years). In all patients, there was a strong association between AH GDF15 and MD on HVF testing (*r* = −0.80, 95% confidence interval [CI], −0.95 to −0.39; *P* = 0.003) (Fig. 3A). This association was similar when examining patients with POAG and PXG separately (POAG: *r* = −0.94; 95% CI, −0.99 to −0.33; *P* = 0.02; PXG: *r* = −0.92; 95% CI, −0.99 to −0.41; *P* = 0.01) (Fig. 3B). We found similar associations with nonparametric Spearman rank correlation coefficients (POAG: ρ = −0.80; PXG: ρ = −0.94), demonstrating the robustness of our results despite the small sample size. Although there were two patients with PXG whose AH GDF15 levels appeared to be distinctly different from the other four patients, Grubbs’ test revealed that they were not outliers at the two-tailed α = 0.05 level. Moreover, the significant association remained even when omitting these two high-leverage data values (*r* = −0.95; ρ = −1.00; Fig. 3C), strengthening the robustness of our findings.

To determine the magnitude of the effect size, we also generated a linear regression model with the independent variable of AH GDF15 and the dependent variable of MD. Increased AH GDF15 was significantly associated with worse MD (β = −0.042; 95% CI, −0.065 to −0.018; *P* = 0.003). When examining patients with POAG and PXG separately, we found similar effect sizes (POAG: β = −0.12; 95% CI, −0.21 to −0.040; *P* = 0.02; PXG: β = −0.050; 95% CI, −0.081 to −0.020; *P* = 0.01). Cumulatively, our findings suggest that AH GDF15 is detectable in PXG and POAG and similarly increases with worse visual field loss in both disease subtypes.

## Discussion

In this study, we found that AH GDF15 is detectable in patients with PXG, similar to our previous observations in patients with POAG. Of interest, we found similar associations between elevations in AH GDF15 and visual field loss in patients with POAG and PXG. These findings suggest that elevations in AH GDF15 are likely associated with worse glaucoma severity and not dependent on the underlying pathophysiology, supporting the idea that GDF15 as a molecular marker could be generalizable to PXG and perhaps other forms of glaucoma.

These findings are significant since they may lead to improved treatment algorithms for PXG, which are needed given its challenging clinical trajectory. One strength of this study was deliberate masking to mitigate the risk of bias. The investigator reviewing the medical record to obtain demographic and clinical information was masked to GDF15 levels, while the investigator measuring AH GDF15 levels was masked to clinical information. Furthermore, our consistent findings in patients with POAG compared to our prior work^7^ attest to the rigor of our study design and analytic approach.

Although we exceeded the sample size necessary by power analysis and found significant associations, our small sample size did not permit us to perform multivariable analysis to control for possible confounding variables, including prior glaucoma surgery. The fact that some patients with PXG had prior glaucoma surgery may have influenced the results of this study. Moreover, in this study, no patients with POAG or PXG had AH GDF15 levels below the limit of detection of commercially available ELISA, while some patients from our previous study did. We speculate that this difference may have arisen due to the worse visual field loss of the patients recruited for this study. Further studies examining the analytical and biological variability of AH GDF15 not only at single time points but also in a longitudinal fashion are essential to further characterize the temporal dynamics of AH GDF15 elevation in relation to RGC stress and ultimate demise. Finally, as is the case for all molecular markers, it will be essential to rigorously examine the sensitivity and specificity of AH GDF15 given that serum GDF15 has been reported as a molecular marker for other systemic diseases, including cardiovascular diseases, obesity, kidney diseases, and diabetes. These future studies are essential before we can fully understand the translational possibilities of GDF15 as a molecular marker for glaucoma.
